# Ultrasound- and Doppler-Guided WALANT Arthroscopic Surgery for Patellar Tendinopathy with Partial Rupture in Elite Athletes—A 2-Year Follow-Up of a Prospective Case Series

**DOI:** 10.3390/medicina60040541

**Published:** 2024-03-27

**Authors:** Håkan Alfredson, David Roberts, Christoph Spang, Markus Waldén

**Affiliations:** 1 Sports Medicine Unit, Department of Community Medicine and Rehabilitation, Umeå University, 90187 Umeå, Sweden; 2 Alfredson Tendon Clinic, Capio Ortho Center Skåne, 21532 Malmö, Sweden; 3 Capio Ortho Center Skåne, 21532 Malmö, Sweden; david.roberts@capio.se (D.R.); markus.walden@telia.com (M.W.); 4 Anatomy Section, Department of Integrative Medical Biology, Umeå University, 90187 Umeå, Sweden; christoph.spang@spine-research.de; 5 Institute for Sports Science, Würzburg University, 97082 Würzburg, Germany; 6 Private Orthopaedic Spine Center, 97080 Würzburg, Germany; 7 Department of Health, Medicine and Caring Sciences, Linköping University, 58183 Linköping, Sweden

**Keywords:** patellar tendinopathy, partial rupture, elite, surgery, follow-up

## Abstract

*Background and Objectives*: Patellar tendinopathy is difficult to treat, and when combined with partial rupture, there are additional challenges. The aim of this study was to evaluate the subjective outcome and return-to-sport status after ultrasound (US)- and colour doppler (CD)-guided wide awake local anaesthetic no tourniquet (WALANT) arthroscopic shaving in elite athletes. *Material and Methods*: Thirty Swedish and international elite athletes (27 males) with a long duration (>1 year) of persistent painful patellar tendinopathy in 35 patellar tendons, not responding to non-surgical treatment, were included. All patients were treated with the same protocol of arthroscopic shaving, including bone removal and debridement of partial rupture, followed by at least 3 months of structured rehabilitation. The VISA-P score and a study-specific questionnaire evaluating physical activity level and subjective satisfaction with the treatment were used for evaluation. *Results*: At the 2-year follow-up (mean 23, range 8–38 months), 25/30 patients (29/35 tendons) were satisfied with the treatment result and had returned to their pre-injury sport. The mean VISA-P score increased from 37 (range 7–69) before surgery to 80 (range 44–100) after surgery (*p* < 0.05). There was one drop-out (one tendon). There were no complications. *Conclusions*: US- and CD-guided WALANT arthroscopic shaving for persistent painful patellar tendinopathy, including bone removal and debridement of partial rupture, followed by structured rehabilitation showed good clinical results in the majority of the elite-level athletes.

## 1. Introduction

Patellar tendinopathy is a relatively common injury in athletes participating in sports with repetitive high and explosive loads on the knee extensor mechanism [1,2,3,4,5]. Typical sports are so-called jumping sports, like volleyball and basketball, but the condition is also seen among athletes involved in football, handball, and rugby [3,4,6], which are sports with multiple rapid accelerations and decelerations. The condition is well-known to be difficult to treat. The first line of treatment is commonly rest and NSAIDs, followed by stretching and specific exercises [1,7]. Different types of loading regimens, such as painful eccentrics and heavy-slow quadriceps training, are considered the best non-surgical treatment methods and often show good clinical results [7,8,9,10]. There is, however, a sub-group of patients, usually high-level athletes, that do not respond to non-surgical treatment for whom surgery is needed. In our experience, patients with patellar tendinopathy combined with partial tendon rupture [11], respond less to non-surgical treatments. It can be difficult to accurately diagnose a partial rupture inside a tendinopathic tendon, but if the ultrasound examination shows bone pathology (spur or prominent bone edge) in the patellar tip, it should be suspected [11]. The final diagnosis of a partial rupture is established during surgery.

The most commonly used surgical method worldwide is open surgery in general or spinal anaesthesia, with intra-tendinous debridement of macroscopically abnormal tendon tissue, combined with a long rehabilitation period of up to 9–12 months [9]. The clinical results have, however, been shown to vary, and this surgical method has been questioned [8]. Around 15 years ago, ultrasound (US)- and colour doppler (CD)-guided wide awake local anaesthetic no tourniquet (WALANT) arthroscopic surgery was introduced. This is a surgical method based on research findings from basic biology showing that the nerves in patellar tendinopathy were located outside the deep (dorsal) side of the tendon [12]. During surgery, the main target is the very localised region with blood vessels and nerves outside the tendon, minimising trauma to the proper tendon. This new method was evaluated in one thesis, one randomised study (compared with sclerosing injection treatment), and in multiple cohort studies and case series, showing good clinical results [6,13,14,15,16,17,18].

This study aimed to evaluate the subjective outcome and return-to-sport status after US- and CD-guided WALANT arthroscopic shaving, including bone removal and revision of partial rupture, in elite athletes with persistent patellar tendinopathy not responding to non-surgical treatment.

## 2. Materials and Methods

### 2.1. Study Sample

**Figure 1:**
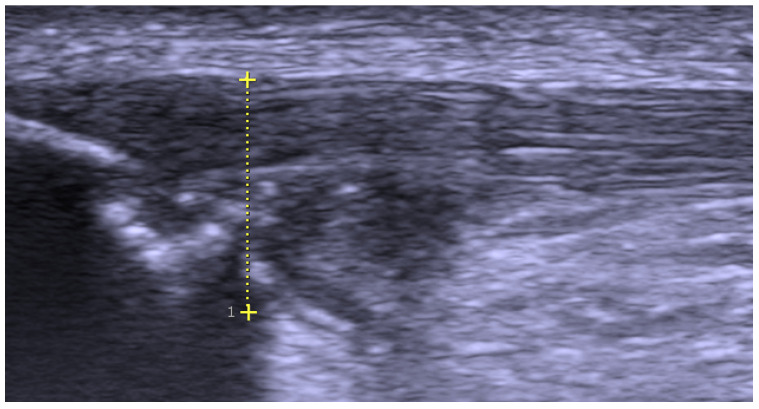
Greyscale ultrasound examination—longitudinal view of a patient with proximal patellar tendinopathy. There is a thickening of the proximal patellar tendon (marked) including tendinopathy on the dorsal side and a suspected partial rupture in close relation to a sharp bone edge in the patellar tip.

Swedish and international elite athletes seeking help at an international tendon clinic for persistent painful patellar tendinopathy between September 2020 and April 2023 were included in this surgical follow-up. All patients were evaluated pre-operatively and operated at the Alfredson Tendon Clinic, Capio Ortho Center Skåne, in Malmö, Sweden. Patients were included if (1) they were 18 years or older, (2) clinical examination and US findings showed proximal patellar tendinopathy (Figure 1), (3) non-surgical management including specific loading regimens was unsuccessful (>6 months), and (4) they had pain on a level where they could not train and compete at the desired level. Patients were excluded if they (1) did not train and compete in professional or elite sports, (2) had previous surgery inside or close to the patella tendon, and (3) had chronic systemic inflammatory conditions.

There were 30 consecutive elite (22 professional and 8 non-professional) athletes (27 males and 3 females) included with 35 tendons treated; staged surgery was performed for patients with bilateral treatments. The mean age in males was 25 (range 18–39) and in females was 28 (range 20–33) years. The athletes were active in different sports including football (*n* = 7), handball (*n* = 6), rugby (*n* = 4), alpine skiing (*n* = 3), ice hockey (*n* = 4), hurling (*n* = 1), triathlon (*n* = 1), tennis (*n* = 1), volleyball (*n* = 1), cross-fit (*n* = 1), and American football (*n* = 1).

Previous non-surgical management for each tendon included loading regimens (*n* = 35), NSAIDs (*n* = 35), shock wave (*n* = 5), PRP injection (*n* = 8), cortisone injection (*n* = 8), sclerosing polidocanol injection (*n* = 4), high volume injection of local anaesthesia + cortisone (*n* = 1), EPI (*n* = 2), and stem cell injection (*n* = 1).

### 2.2. Pre-Operative Examination

All patients were examined clinically by one (HA) or two experienced orthopaedic surgeons (HA and MW) with a standard knee joint examination ruling out other pathology such as patellofemoral complaints, bursitis, fat pad syndrome, etc., and verifying tenderness on the proximal pole of the patella tendon. Immediately following the clinical examination, patients were also examined bedside with a high-resolution greyscale US + CD (S-500, Siemens AG, Munich, Germany) using a linear multifrequency (8–13 MHz) probe, confirming structural changes typical for patellar tendinopathy. In general, scanning showed a thickened proximal patellar tendon of varying size (>6 mm) with irregular structure mainly dorsal and central in the tendon, including focal hypo-echoic regions and high blood flow coming from the dorsal soft tissues and going into the tendon [4,10]. In all patients, there were bony prominences (spur, sharp edge, separate loose fragments, etc.) in and below the patellar tip. There was also regularly a local thickening of the paratenon, including high blood flow, on the superficial side of the proximal patellar tendon [19].

### 2.3. Surgical Treatment

The surgical treatment was performed in local anaesthesia exclusively by one (HA) or two (HA and MW) orthopaedic surgeons [6,11,19,20]. After disinfecting the skin with wet cloths of chlorhexidine cutaneous solution (Klorhexidinsprit 5 mg/mL, Fresenius Kabi AB, Uppsala, Sweden), 5 mL of a local anaesthetic (Xylocain + adrenalin 10 mg/mL + 5 μg/mL, Aspen, Durban, South Africa) was injected in the skin for each of two standard anterolateral and anteromedial portals followed by 20 mL of another local anaesthetic (Carbocain + adrenalin 10 mg/mL + 5 μg/mL, Aspen, South Africa) in the knee joint. The skin was then scrubbed and draped with a sterile paper-cover exposing only the knee joint from the middle of the thigh to the tibial tuberosity.

**Figure 2:**
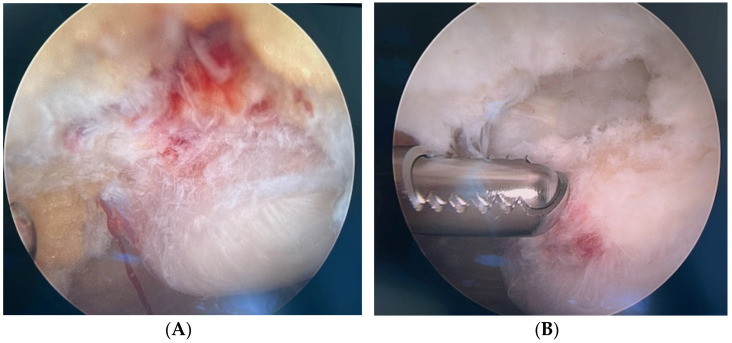
Pictures from surgery. (**A**). A richly vascularised fatty infiltration is seen on the dorsal side of the proximal patellar tendon. (**B**). After the removal of prominent bone, a partial rupture is identified on the dorsal side of the proximal patellar tendon.

The patients were operated on in a supine position with a straight knee and relaxed quadriceps without a tourniquet. We used a pressure-controlled pump (Arthrex, Naples, FL, USA). A standard arthroscopic evaluation of the whole knee joint was performed. In the case of an obliterating suprapatellar or a prominent medial plica, these were routinely resected. Then, the patella tendon insertion into the patella was identified, and a 4.5 mm full radius blade shaver (Arthrex, Naples, FL, USA) was introduced. Simultaneous per-operative US examination (longitudinal and transversal views) guided the procedure. Careful shaving, aiming to destroy only the region with high blood flow and nerves adjacent to the tendinosis changes on the dorsal side of the tendon (separating the Hoffa fat pad from the patellar tendon), was performed. Extensive fat pad resection was avoided. In cases with a bone spur/sharp edge in or a loose bone fragment below the patellar tip, careful removal with the shaver and a ball end mill (Arthrex, Naples, FL, USA) was performed. It was often necessary to remove some tendinosis tissue and poor-quality ruptured tissue to reach the sharp bone, but emphasis was put on preserving tendon tissue. In direct relation to these patellar bony prominences, there were always partial tendon ruptures of varying sizes, appearing as a painful hole in the tendon with often richly vascularised walls (Figure 2A,B). The partial tendon rupture was debrided until fresh borders. Sometimes, 3–5 mL of additional local anaesthesia (Xylocain + adrenalin 10 mg/mL + 5 μg/mL, Aspen, South Africa) was needed to be injected into the site for the partial rupture allowing for a continued pain-free procedure. The portals were closed using single non-resorbable sutures, which were removed after one week, and 20 mL of a long-acting local anaesthetic (Ropivacain 10 mg/mL, Fresenius Kabi AB, Sweden) was injected into the joint.

After the arthroscopy, there was a minor open procedure on the superficial side of the proximal patellar tendon. Preceded by 3–5 mL of local anaesthesia (Xylocain + adrenalin 10 mg/mL + 5 μg/mL, Aspen, South Africa), and via a short longitudinal skin incision the prepatellar bursa and paratenon was opened and regions with richly vascularised infiltrative fat tissue were scraped away from the tendon surface [20]. The wound was closed with subcutaneous resorbable sutures and single non-resorbable sutures in the skin. A sterile wound dressing was applied with an elastic bandage from the toes to above the knee. No pre-operative antibiotics or post-operative thrombosis prophylaxis was used.

### 2.4. Post-Operative Care and Rehabilitation

Patients were supplied with a pain-killer bag consisting of Paracetamol (500 mg, 8 tablets), Diclofenac (50 mg, 2 tablets), and Oxycontin. They then rested overnight at home or at a nearby hotel with partial weight bearing using crutches and assisted leg lifts allowed, and a majority were seen for a follow-up visit the day after surgery. If the knee joint was swollen with significant pain and/or restricted range of motion, hemarthrosis evacuation was performed under strict sterile conditions.

For Swedish patients, follow-ups with clinical examination and US + CD scanning were scheduled for 3, 6, 12, 16, 26, and 52 weeks after surgery. For international patients, there was only the follow-up visit the day after surgery and then they were followed via mail or telephone with the player, the referring physician, or the team physiotherapist.

All patients followed the same principal rehabilitation program (Table 1). In the first 4 weeks, there was only light loading allowed to ensure that the tissue defects would fill. For strength training, only closed-chain exercises with planted foot were used.

### 2.5. Outcome Measures

The self-administered Victorian Institute of Sports Assessment Patella (VISA-P) functional score and return-to-pre-injury sport were used for evaluation. The VISA-P score was completed by the patients on the day of surgery (pre-treatment) and at follow-up. For the Swedish patients, a study-specific questionnaire evaluating subjective satisfaction with the treatment (satisfied or not satisfied) and return-to-pre-injury sport was completed at the final follow-up. In a sub-group of international athletes (*n* = 6, 8 tendons), follow-up was restricted to evaluating return-to-pre-injury sport exclusively, with no VISA-P scoring.

### 2.6. Ethics

This study was conducted in accordance with the Declaration of Helsinki and approved by the Swedish Ethical Review Authority (reference number 2022-06-28-02889-01).

### 2.7. Statistical Methods

SPSS (Statistical Package of Social Science, Version 28) was used to analyse the data (SPSS Inc., Chicago, IL, USA). All calculations were measured on a group level with a paired *t*-test to analyse whether there were differences before and after surgery. The significance level was set to *p* < 0.05.

## 3. Results

At the follow-up at a mean of 23 months (range 8–38) after the operation, 25/30 patients (29/35 tendons) had returned to their pre-injury sport. The mean VISA-P functional score for the whole group increased significantly from 37 (range 7–69) before surgery to 80 (range 44–100) (*p* < 0.05) after surgery.

For the Swedish patients, 14/15 (17/18 tendons) provided VISA-P follow-up scores and were satisfied with the treatment result. One Swedish patient did not complete the VISA-P after surgery but responded via telephone to subjective satisfaction (being satisfied) and return-to-sport status (had returned to pre-injury professional sport). For the international patients, we received VISA-P follow-up scores from 8/15, and for another 6/15, we obtained information about the return to their pre-injury sport. Twelve of the international patients (13 tendons) had returned to their pre-injury sports. For one international patient, no 1-year information at all could be retrieved (drop-out).

In the analysis of the four patients (five tendons) who were not satisfied with the treatment, one patient (two tendons) had retired from their pre-injury sport due to associated widespread grade 3–4 cartilage lesions in the patellofemoral joint of both knees. The second patient retired from pre-injury sport after a re-operation, also because of grade 4 cartilage lesions in the patellofemoral joint. The third patient had remaining tendon-related pain and retired from pre-injury sport. The final patient had remaining tendon-related pain and had not been able to return to pre-injury sport but was participating at a lower level and had not decided to retire.

There were no complications in relation to the surgery including no superficial or deep surgical site infections and no thromboembolic events.

## 4. Discussion

The principal finding in this prospective case series on elite athletes suffering from persistent painful proximal patellar tendinopathy, including bony pathology and partial rupture, and operated with the US-guided arthroscopic shaving procedure, was a favourable outcome with significant improvement in VISA-P, high patient satisfaction, and high return rate to pre-injury professional and elite sports.

### 4.1. Subjective Improvement and Return to Sport

The aetiology of patellar tendinopathy is generally considered to be overloading, possibly influenced by anthropometric factors [5]. It is a relatively common condition among athletes involved in leg explosive sports, and the pain related to this condition often leads to decreased maximal performance and sometimes retirement from their sport [2]. Jumping sports like volleyball and basketball have dominated, but lately, also other explosive sports have been shown to be affected by this troublesome condition. All patients in the current study were active in heavy patellar tendon-loading sports, where most were football, handball, and rugby players. Could it be that the partly changed characteristics of these sports, focusing on speed and explosiveness, increase the risk of getting patellar tendinopathy? The current study showed a favourable group outcome with high patient satisfaction, significant improvement in VISA-P scores, and a high return-to-sport rate. In fact, all patients improved in their VISA-P scores following surgery and rehabilitation, and all but four patients (five tendons) were satisfied with the treatment and had returned to their pre-injury sport at the same level as before symptom onset. In most cases, patients improved, but were not cured; however, nine patients scored more than 90 on the VISA-P, indicating that they had negligible or only slight remaining symptoms.

### 4.2. Bony Pathology and Partial Tendon Rupture

All patients in our study had been treated with different types of loading regimens for a long period and had usually been kept in training and competition with this regimen. In the history taken from the patients, a frequent comment was that there was a sudden worsening of the pain, where this “new” pain was on a level where the loading regimen was not enough to keep them in their sport anymore. Theoretically, this could likely be the transition when they sustained the partial tendon rupture. In a previous study, we showed that when US showed bony pathology (bone spurs, sharp edges, loose fragments, etc.) in the patellar tip, there was a high frequency of associated partial rupture on the deep side together with the tendinopathy [11]. In the current study on elite athletes exclusively, all patients had pre-operative US findings showing bony pathology, and all patients also had partial ruptures identified during surgery. This novel finding is clinically relevant for the surgeon and strengthens the indication to carefully explore the tendon near the bony pathology. In our experience, it is important to find these partial ruptures because we strongly believe that they are related to the worsened pain during tendon loading, especially with an “acute-on-chronic” onset. With that said, we have also noticed that the original intra-articular local anaesthesia is sometimes not enough when opening up the site for a partial rupture with the shaver, and further probing of the partial rupture walls causes sharp pain. Then, additional local anaesthesia injected into the site for the partial rupture allows for a continued pain-free procedure. This temporary pain response in the fully awake patient thus guides the surgery and is a strong argument for performing WALANT surgery. We have many years of experience in performing this surgery in local anaesthesia exclusively and have never had to convert to general anaesthesia because of pain or discomfort [6,11,13,14,15,16,17,19,20].

The high numbers of partial ruptures among our patients are not representative of the traditional patient with patellar tendinopathy. The reason for this is likely that most patients in our study represent second opinion and so-called “end-stage cases”. Altogether, there seems to be a wide spectrum of patients, from those having “isolated” tendinopathy to others having tendinopathy plus bony pathology and partial tendon rupture.

### 4.3. Complications

The surgical procedure with shaving of the deep side of the patellar tendon, removal of sharp bone in the patellar tip, and debriding partial ruptures to normal borders, was performed in all patients without complications. Occasionally, there is significant early hemarthrosis resulting in pain and restricted range of motion, and evacuation the day after surgery is sometimes needed for the patient to be able to comfortably start to load with full weight-bearing walking. There were no superficial or deep surgical site infections and no thromboembolic events despite no per-operative antibiotic prophylaxis or post-operative thrombosis prophylaxis.

### 4.4. Methodological Considerations

First, randomised controlled trials are preferred when designing studies on new treatment methods, but it is well-known that professional/elite athletes are difficult to recruit and include in such trials. Therefore, an observational study design using both subjective and objective outcome data, as in this study, might be more suitable when reporting clinical results for this specific patient sub-group.

Second, the study sample of 30 Swedish and international elite athletes with 35 painful tendons is limited, but we only included professional and elite-level amateur athletes. Our clinic is, however, the only centre in the county of Skåne (population of approximately 1.5 million citizens) where patients with patellar tendinopathy are operated, and we recruited patients consecutively for 2.5 years. During this period, there were also additional Swedish recreational/amateur athletes on a non-elite level who were operated with the same surgical procedure. These patients were, however, excluded from this report, and the findings in the current study cannot be generalised to such patients. Additionally, there were only three female patients in our series, and we therefore refrained from reporting sex-specific results; consequently, some caution is recommended when interpreting the findings for females.

Third, we used the VISA-P functional score, which has been shown to be reliable and suitable to monitor the symptoms in athletes [21]. The low scores before surgery despite long non-surgical treatment strengthened the indication for surgical treatment. A weakness is, however, that it was not possible to follow up all the international patients using VISA-P scores even though this is a well-known problem when international professional athletes are involved in studies [21].

Fourth, we decided to use the return to their pre-injury sport at the same level as before symptom onset as the definition for a good result. Unfortunately, we do not know the exact length of absence for most patients after surgery, but the earliest known return was 12 weeks after surgery. There is, however, most likely a wide range in terms of clearance for return to sports among athletes and among different sports.

Fifth, although the main focus was on the patient selection and surgical procedure itself, the post-operative rehabilitation protocol is likely also of utmost importance. Great care is taken to minimise the removal of fat in the Hoffa fat pad and peri-tendon/tendon tissue. This is achieved by using ultrasound guidance. Filling of the defects usually takes approximately four weeks. During this period, rehabilitation needs to be slow and careful to prevent the creation of persistent fluid accumulation beneath the tendon. We, therefore, routinely recommend US scanning 3–4 weeks after surgery to guide the introduction and progression of concentric and eccentric strength training of the quadriceps. Importantly, the athlete should be able to perform one-legged eccentric quadriceps exercises with high loads, depending on the sport’s requirement, before plyometrics is introduced.

When looking at previous treatments, all patients in the current study had been treated with loading regimens and NSAIDs. An interesting observation was that among the international patients, it was more common with injection treatments such as PRP, Cortisone, and even stem cells, while among the Swedish patients, it was more common with shock wave treatment.

## 5. Conclusions

In conclusion, for professional and elite athletes suffering from persistent painful patellar tendinopathy, US- and CD-guided WALANT arthroscopic shaving, including bone removal and debridement of partial rupture, showed good clinical results and no complications.

## Figures and Tables

**Table 1:** General guidelines for introduction and progression of activities and rehabilitation exercises.

Week	Rehabilitation Activities and Exercises
1–2	Gradually increased full weight-bearing walking, full range of motion exercises and isometric quadriceps contraction exercises. Short sessions with light-load biking.
3–4	Increased walking distances and light-load biking distances. Isometric quadriceps exercises.
5–6	Introduction of closed chain concentric quadriceps strength training (low-load and multiple repetitions). Introduction of two-legged low-load eccentric exercises. Free walking distances and heavier biking (including resistance intervals).
7–8	Heavier closed-chain concentric and two-legged eccentric quadriceps exercises Free biking.
9–10	Heavy concentric exercises and introduction of one-legged eccentric exercises.
11	Careful introduction of plyometric exercises (if tolerate heavy isometric, concentric and eccentric strength training).
12	Introduction of sport-specific exercises.

## Data Availability

The data presented in this study are available upon request from the corresponding author (accurately indicate status).
